# Immunogenicity of Mix-and-Match CoronaVac/BNT162b2 Regimen versus Homologous CoronaVac/CoronaVac Vaccination: A Single-Blinded, Randomized, Parallel Group Superiority Trial

**DOI:** 10.3390/vaccines11081329

**Published:** 2023-08-05

**Authors:** Samar Samoud, Jihene Bettaieb, Mariem Gdoura, Ghassen Kharroubi, Feriel Ben Ghachem, Imen Zamali, Ahlem Ben Hmid, Sadok Salem, Ahmed Adel Gereisha, Mongi Dellagi, Nahed Hogga, Adel Gharbi, Amor Baccouche, Manel Gharbi, Chadha Khemissi, Ghada Akili, Wissem Slama, Nabila Chaieb, Yousr Galai, Hechmi Louzir, Henda Triki, Melika Ben Ahmed

**Affiliations:** 1 Department of Clinical Immunology, Pasteur Institute of Tunis, Tunis 1002, Tunisia; samar.samoud@pasteur.tn (S.S.); imen.zamali@fmt.utm.tn (I.Z.); ahlem.benhmid@pasteur.tn (A.B.H.); gereisha.ahmedadel01@gmail.com (A.A.G.); yousr.galai@pasteur.tn (Y.G.); hechmi.louzir@pasteur.tn (H.L.); 2 Faculty of Medicine of Sousse, University of Sousse, Sousse 4000, Tunisia; 3 Department of Medical Epidemiology, Pasteur Institute of Tunis, Tunis 1002, Tunisia; jihene.bettaieb@pasteur.tn (J.B.); ghassen.kharroubi@pasteur.tn (G.K.); sadok-salem@live.fr (S.S.); dellagi.mongi@gmail.com (M.D.); adel.gharbi@pasteur.tn (A.G.); amorelarbibaccouche@gmail.com (A.B.); 4 Faculty of Medicine of Tunis, University of Tunis, Tunis 1002, Tunisia; 5 Laboratory of Transmission, Control and Immunobiology of Infections (LR11IPT02), Institut Pasteur de Tunis, University of Tunis El Manar, Tunis 1068, Tunisia; henda.triki@pasteur.tn; 6 Department of Clinical Virology, Pasteur Institute of Tunis, Tunis 1002, Tunisia; mariem.gdoura@pasteur.tn (M.G.); nahed.hogga@pasteur.tn (N.H.); gmanel599@gmail.com (M.G.); chadha.khemissi@etudiant-esstst.utm.tn (C.K.); 7 Faculty of Pharmacy, University of Monastir, Monastir 5000, Tunisia; 8 Vaccination Center of Ariana City, Ariana Regional Health Directorate, Ariana 2080, Tunisia; feriel.ferchiou@yahoo.fr (F.B.G.); ghada.elakili1@gmail.com (G.A.); slamawissem02@gmail.com (W.S.); nabilachaieb71@gmail.com (N.C.)

**Keywords:** COVID-19, BNT162b2, CoronaVac, immunogenicity, tolerance

## Abstract

(1) Background: This study aimed to compare the immunogenicity of the mix-and-match CoronaVac/BNT162b2 vaccination to the homologous CoronaVac/CoronaVac regimen. (2) Methods: We conducted a simple-blinded randomized superiority trial to measure SARS-CoV-2 neutralization antibodies and anti-spike receptor binding domain (RBD) IgG concentrations in blood samples of participants who had received the first dose of CoronaVac vaccine followed by a dose of BNT162b2 or CoronaVac vaccine. The primary endpoint for immunogenicity was the serum-neutralizing antibody level with a percentage of inhibition at 90% at 21–35 days after the boost. A difference of 25% between groups was considered clinically relevant. (3) Results: Among the 240 eligible participants, the primary endpoint data were available for 100 participants randomly allocated to the mix-and-match group versus 99 participants randomly allocated to the homologous dose group. The mix-and-match regimen elicited significantly higher levels of neutralizing antibodies (median level of 96%, interquartile range (IQR) (95–97) versus median level of 94%, IQR (81–96) and anti-spike IgG antibodies (median level of 13,460, IQR (2557–29,930) versus median level of 1190, IQR (347–4964) compared to the homologous group. Accordingly, the percentage of subjects with a percentage of neutralizing antibodies > 90% was significantly higher in the mix-and-match group (90.0%) versus the homologous (60.6%). Interestingly, no severe events were reported within 30 days after the second dose of vaccination in both groups. (4) Conclusions: Our data showed the superiority of the mix-and-match CoronaVac/BNT162b2 vaccination compared to the CoronaVac/CoronaVac regimen in terms of immunogenicity, thus constituting a proof-of-concept study supporting the use of inactivated vaccines in a mix-and-match strategy while ensuring good immunogenicity and safety.

## 1. Introduction

The COVID-19 pandemic has exposed the shortcomings of health systems around the world, particularly regarding preventive health. Even though several vaccines have been developed, especially those carried out from new platforms in record time, access to the vaccine has not been equitable between all countries.

In Tunisia, like in other low and middle-income countries, the national strategy vaccination campaign was faced with the need to use different vaccines according to the market availability and also donations from supportive countries and international organizations [1].

At the time of the study, several vaccines have obtained their marketing authorization in Tunisia and are, therefore, widely used [1]. By 22 September 2022, 6,384,538 citizens, i.e., 53.13% of the general population, have been considered to be fully vaccinated according to current international recommendations [1]. More specifically, seven vaccines were available, namely, mRNA-1273 or Spikevax (Moderna), BNT162B2 or Comirnaty (Pfizer-BioNTech), Gam-COVID-Vac or Sputnik V (Gamaleya Research Institute), ChAdOx1-S or Vaxzevria (AstraZeneca), BIBP (Sinopharm), CoronaVac (Sinovac), and Janssen vaccine (Ad26COV2.S). However, these vaccines seem to show relatively heterogeneous efficacies [2,3]. CoronaVac (Sinovac Research and Development Co), an inactivated whole-virion SARS-CoV-2 vaccine, for instance, appears to have an efficacy that varies between sites from 51% to 83% according to phase 3 data [4,5,6]. Although in the latest real-life study in Chile, its efficacy in protecting against symptomatic forms was 87.5%, the immunogenicity of this vaccine appeared to be lower than that of RNA or adenovirus vaccines or that conferred by a previous infection [7,8,9].

Given the disparity between published studies’ results on the effectiveness of inactivated virus vaccines and the effective vaccination of a part of the population by the latter, particularly vulnerable ones, either by choice or by necessity (vaccine scarcity), it was interesting to do a pioneer clinical trial including naive COVID-19 vaccine subjects in order to have relevant data allowing health policymakers to implement the vaccine strategy. Our trial takes on all its importance with the continuing threat of the emergence of new variants that confront us with two challenges: the effectiveness of the immune response and the duration of the conferred protection. Both seem to differ depending on the vaccine’s type, deriving from traditional or new platforms.

Given that several recent studies show the superiority of the “mix-and-match” vaccination protocol [10], in particular, Pfizer/AstraZeneca or AstraZeneca/Pfizer compared to the homologous protocol [11,12,13], we propose herein to compare the immunogenicity of two doses of CoronaVac to that of a first dose of CoronaVac followed by a second shot with the mRNA-based BNT162b2 SARS-CoV-2 vaccine (Comirnaty, Pfizer-BioNTech).

## 2. Methods

### 2.1. Study Design

We conducted a single-blinded, randomized, controlled superiority trial in terms of immunogenicity of the mix-and-match immunization with BNT162b2 versus homologous immunization with the CoronaVac vaccine. Both groups received CoronaVac as the first vaccine dose.

### 2.2. Ethics Statement

The study protocol was approved by the Tunisian National Committee for the Protection of Persons. The trial was registered under the reference numbers: TN2021-NAT-INS-70 and NCT05668065. All participants had adequate time to understand the study and voluntarily dated and signed the informed consent form. They reserved the right to withdraw from the study at any time.

### 2.3. Selection Criteria

All consenting adults aged between 18 and 60 years old with no or well-controlled comorbidities, excluding any immune deficiency disease or disorder, any disability (mainly mental disabilities), and without any history of symptoms suggestive of COVID-19 or a positive COVID-19 test were considered eligible for the study.

The presence of contraindication to any of the vaccines used, pregnancy, or intent to conceive were considered as non-inclusion criteria.

Moreover, the occurrence of a serious adverse event (death, anaphylactic shock, etc.), wishing to withdraw from the study, and the occurrence of a SARS-CoV-2 symptomatic infection during the follow-up period were also considered as exclusion criteria.

### 2.4. Study Design

The study was a simple-blinded, randomized, superiority comparative clinical trial including two parallel arms:

- Arm A:
  - 1st dose: CoronaVac
  - 2nd dose at 21 ± 3 days: CoronaVac
- Arm B:
  - 1st dose: CoronaVac
  - 2nd dose at 21 D ± 3 D: BNT162b2
- Hypothesis (unilateral test):
  - H0: PA = PB
  - H1: PA < PB

PA and PB are the proportion of vaccines with neutralizing antibodies giving 90% inhibition measured after 3 to 5 weeks of the second dose.

### 2.5. Randomization and Masking

All volunteers meeting trial criteria were randomly assigned to receive CoronaVac or BNT162b2 on the day of the second dose. Participants were block-randomized (block size six, ratio of 1:1) to the heterologous second dose arm or to the homologous one. The randomization list was prepared by the study methodologist using a random number table.

Both participants and laboratory staff carrying out the formal analysis were blinded to the second dose of vaccination.

### 2.6. Procedures

Study participants were recruited from four different sites located in the North of Tunisia: the vaccination center of Ariana, Géant supermarket (Governorate of Ariana), Leoni factory (Governorate of Bizerte), and STEG (Tunisian Electricity and Gas Company, head office, Governorate of Tunis). After obtaining the informed consent, all included subjects were interviewed face-to-face by trained interviewers in order to complete a paper questionnaire.

All participants then received CoronaVac as the first vaccine dose consisting of 0.5 mL containing 600 SU in-house units (equal to 3 μg) of SARS-CoV-2 antigen. All the subjects were invited to receive the second dose three weeks later. To minimize bias, randomization was delayed till the day of the second dose. Then, one arm was assigned to receive CoronaVac as a second dose, and one arm received the BNT162b2 vaccine consisting of a 0.3 mL prefilled syringe containing 30 µg of mRNA encoding the viral spike (S) glycoprotein of SARS-CoV-2.

A blood sample (5 mL of whole blood) was collected for each participant at the vaccination location (center or company) before the first dose injection. After verifying the absence of a SARS-CoV-2 infection by PCR during the follow-up survey, a 2nd and a 3rd blood sample (5 mL of whole blood each) were collected for each participant at the vaccination location before the second dose injection and between day 21 and day 35 after the second dose, respectively. Data collection was treated at the Institute Pasteur of Tunis.

The occurrences of adverse events within the first 7 days after the prime and up to 30 days after the boost vaccination were monitored.

### 2.7. Peripheral SARS-CoV-2 Neutralizing Antibody Measurement

All sera were tested by the cPass^TM^ SARS-CoV-2 Neutralization Antibody Detection Kit (GenScript^®^, Piscataway, NJ, USA). This is a manual ELISA test detecting the antibodies that inhibit the interaction between two recombinant proteins: RBD-HPR of the SARS-CoV-2 wild strain and the ACE2. The assay was performed as below, according to the manufacturer’s instructions. Briefly, the samples and controls are pre-incubated with the HRP-RBD to allow the binding of the circulating neutralization antibodies to HRP-RBD. The mixture is then added to the capture plate, which is pre-coated with the hACE2 protein. The unbound HRP-RBD, as well as any HRP-RBD bound to non-neutralizing antibodies, will be captured on the plate, while the circulating neutralization antibodies-HRP-RBD complexes remain in the supernatant and get removed during washing. After the washing steps, TMB solution is added, making the color blue. By adding Stop Solution, the reaction is quenched, and the color turns yellow. This final solution can be read at 450 nm in a microtiter plate reader.

This test is the first commercial assay that was suggested to substitute the seroneutralization tests on viral cultures [14,15]. It was validated by comparison to the Plaque Reduction Neutralization Test (PRNT) using the SARS-CoV-2 virus WA01/2020 isolate. The positive predictive value was 95·7% (CI 95% [85.8–98.8]), and the negative predictive value was 97.8% (CI 95% [92.5–99.4]). This culture-free test obtained an FDA-EUA for research use only on 11 June 2020. According to the manufacturer, the calculation of the results is as follows:

A cut-off of 30% inhibition of RBD-ACE2 binding was used to determine the presence of neutralizing antibodies.

$$\%\;Signal\;Inhibition=\left(1-\left(\frac{OD\;value\;ofsample}{OD\;value\;of\;negative\;control}\right)\right)\times 100$$

### 2.8. Peripheral Anti-S-RBD and Anti-N Antibody Measurement

Total anti-S-RBD (Receptor Binging Domain) antibodies were quantified by the commercial test Elecsys^®^ Anti-SARS-CoV-2 S (Cat number 09203095190, Roche^®^ Diagnostic, Basel, Switzerland) on the Cobas^®^ e411 analyzer. This quantitative test is calibrated against the 1st WHO international standard 20/136 from the National Institute for Biological Standards and Control, UK. The obtained results are expressed by IU/mL, which corresponds to 0.972 × Binding Antibody Unit per mL. The sensitivity cut-off is equal to 0.8 IU/mL, indicating a previous contact with the virus, and the neutralizing antibody cut-off, equal to 15 IU/mL, indicates the presence of neutralizing antibodies with a positive predictive value of 100%, according to the manufacturer.

Sera were also tested for the detection of the total anti-N specific antibodies by the commercial test Elecsys^®^ anti-SARS-CoV2 qualitative assay (Cat number 09289267119, Roche^®^ Diagnostic, Basel, Switzerland) on the Cobas^®^ e411 analyzer. This test is a qualitative assay with results expressed as an index (reference value < 1).

### 2.9. Outcomes

This trial aimed to test the superiority in terms of immunogenicity of the heterologous mix-and-match immunization with mRNA vaccine (BNT162b2) after vaccination with an inactivated vaccine (CoronaVac) versus homologous immunization with the inactivated vaccine (CoronaVac).

The primary endpoint for immunogenicity was the serum-neutralizing antibody level measured by the percentage of inhibition at 21–35 days after the second dose. A difference of 25% between groups in the proportion of vaccines with a percentage of inhibition at 90% or above was considered clinically relevant.

The secondary outcomes included:

- The anti-spike IgG antibody response at 21–35 days after the second dose
- The immunogenicity parameters (anti-spike neutralizing antibody and IgG antibody titers) at day 0 (at baseline)
- The immunogenicity parameters (anti-spike neutralizing antibody and IgG antibody titers) at day 21 ± 3 days (after the second dose)
- The safety indexes of adverse reactions at day 30.

### 2.10. Statistical Analysis

The sample size was computed using the power and sample size program [version 3.1.6, Window dichotomous]. Assuming a difference of 25% between groups, an alpha of 5%, a power of 90%, and accounting for a 20% loss to follow-up, 96 persons are required to complete the sample size for each arm.

The primary analysis population involved subjects who were randomly assigned, received the second dose, and provided post-boost vaccination data even exceeding the time frame of 21 or 35 days from the booster as fixed by the study protocol.

Graphs and statistical analyses were performed using GraphPad Prism software (version 5.0).

Chi-square tests and *t*-tests were used to test differences between the two groups. Antibody levels were compared using Mann–Whitney tests. Statistical significance was set at *p*-values less than 0.05.

## 3. Results

**Figure 1:**
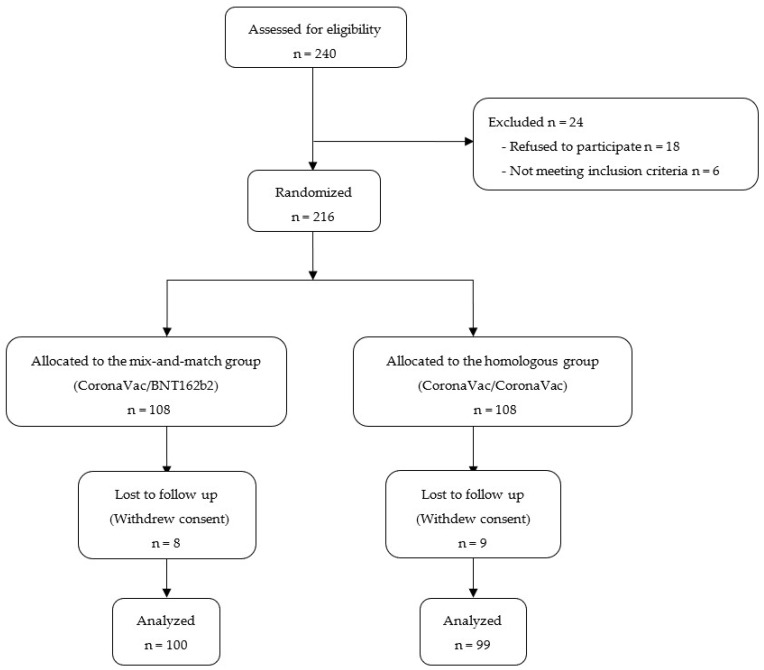
Flow of participants through the trial.

Between November 2021 and April 2022, 240 participants were assessed for eligibility, among whom 6 were excluded for not meeting inclusion criteria, and 18 declined participation after initially consenting to enroll and receiving the first dose of vaccine, yet prior to randomization (Figure 1).

Of the 216 participants who underwent randomization, 17 (7.8%) were lost to follow-up, but there were no significant differences in sex and age between those analyzed and those lost to follow-up. Primary endpoint data were available for 100 participants randomly allocated to the mix-and-match group versus 99 participants randomly allocated to the homologous second dose group (Figure 1).

As summarized in Table 1, the characteristics of participants at baseline were well balanced between homologous and mix-and-match vaccinated groups across conditions. No significant differences were noted in all tested parameters.

**Figure 2:**
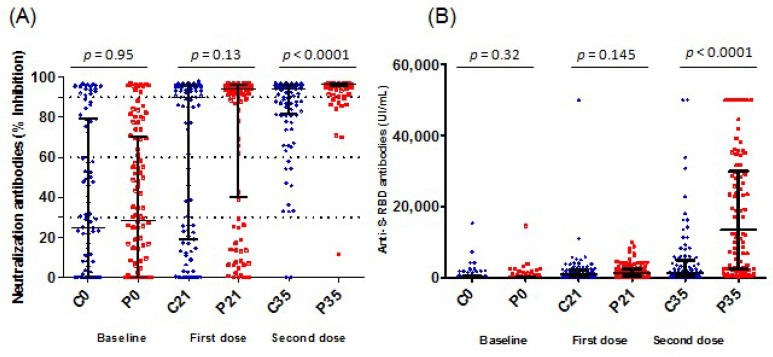
Antibody titers by booster vaccine allocation. The whiskers denote the median with the interquartile range. The comparisons were made using the Mann–Whitney U test. C0: CoronaVac boost group at baseline, C21: CoronaVac boost group after a median of 21 days from first dose, C35: CoronaVac boost group after a median of 35 days from second dose, P0: Pfizer boost group at baseline, P21: Pfizer (BNT162b2) boost group after a median of 21 days from first dose, P35: Pfizer (BNT162b2) boost group after a median of 35 days from second dose. (**A**) SARS-CoV-2 neutralization antibody titers. Measurements by GenScriptcPass^TM^ SARS-CoV-2 neutralization antibody Detection Kit. The dotted lines show limits between low, medium, and high titers. (**B**) SARS-CoV-2 anti-S-RBD IgG titers. Measurements by the commercial test Elecsys^®^ Anti-SARS-CoV-2 S (Roche^®^ Diagnostic, Basel, Switzerland) on the Cobas^®^ e411 analyzer.

As shown in Table 2 and Figure 2 and as expected, no difference was reported between both groups regarding the level of neutralization antibodies and anti-spike IgG antibody responses after the first dose of vaccination. However, after the second dose of vaccination, we noted significantly higher levels of neutralizing antibodies (median level of 96%, IQR [95–97] versus median level of 94%, IQR [81–96]) (*p* < 0.0001) and anti-spike IgG antibodies (median level of 13, 5460, IQR (2557–29,930) versus median level of 1190, IQR (347–4964) in the group who received the BNT162b2 vaccine compared to this who received CoronaVac vaccine (*p* < 0.0001). In the second step, participants were categorized into four subgroups according to the percentage of neutralizing antibodies (30%, 30–60%, 60–90%, and >90%) (Table 2). Interestingly, the percentage of subjects with a percentage of neutralizing 8 antibodies> 90% was 90% in the group who received a mix-and-match regimen versus 60.6% in the group who received a homologous regimen (*p* < 0.0001) (Table 2). Similarly, 78.0% of the subjects from the mix-and-match group exhibited levels of anti-S-RBD antibodies >2500 IU/mL versus 34.3% from the homologous group (*p* < 0.0001).

Regarding the side effects that occurred within 30 days after the second dose of vaccination, no severe events (grade 3) were noted in our study in both groups. However, side effects were significantly more frequent in the CoronaVac/BNT162b2 group compared to the CoronaVac/CoronaVac group (58% versus 32.2%, *p* < 0.0001) (Table 3). More particularly, arm soreness, fever, and headache occurred more frequently in the mix-and-match regimen group (*p* = 0.006, *p* = 0.05, *p* = 0.005, respectively) (Table 3).

## 4. Discussion

Different vaccination strategies were deployed worldwide to control the COVID-19 pandemic. In Tunisia, the general population received different types of vaccines, including inactivated ones in addition to RNA and adenovirus vaccines, according to market availability and also donations from supportive countries. Despite the reported superiority of RNA vaccines for the induction of a specific immune response compared to inactivated virus vaccines [1,16,17], the use of inactivated vaccines was justified in several countries by their availability at times when other alternatives were not accessible yet. Several studies reporting better subsequent immunogenicity with an mRNA vaccine boost after two initial inactivated vaccine doses [18,19,20,21,22,23] have opened up the possibility of offering this vaccination schedule in order to make up for the lack of immunogenicity of the first vaccination. However, little data are available about the inactivated/mRNA vaccine mix-and-match regimen for the first and second doses of COVID-19 vaccination.

To our knowledge, no trial tested the superiority in terms of immunogenicity of the mix-and-match immunization with mRNA vaccine for the second dose after vaccination with an inactivated vaccine for the first dose versus homologous immunization with inactivated vaccines for both doses. Our present trial is a single-blinded, randomized trial ensuring the comparability in terms of immunogenicity of two groups receiving homologous (CoronaVac/CoronaVac) or heterologous (CoronaVac/BNT162b2) primary regimen vaccination, thus constituting a proof-of-concept study.

As expected, no difference was reported between both groups regarding the level of neutralizing antibodies and anti-spike IgG antibody responses after the first dose of vaccination. However, after the second dose of vaccination, significantly higher levels of neutralizing antibodies and anti-spike IgG antibodies were noted in the group who received the BNT162b2 vaccine compared to the one who received the CoronaVac vaccine. Moreover, the percentage of subjects with neutralizing antibody inhibition > 90% was higher than 90% in the group who received a mix-and-match regimen versus 60·6% in the group who received a homologous regimen. Thus, the difference between groups in the proportion of vaccines with a percentage of inhibition at 90% or above was superior to 25%, pointing to the superiority in terms of immunogenicity of the mix-and-match regimen (primary outcome). Similarly, 82% of the subjects who received the BNT162b2 vaccine exhibited levels of anti-S-RBD antibodies >2500 IU/mL versus 34% of the subjects who received CoronaVac. This is not only a proof-of-concept for the superiority of the CoronaVac/BNT162b2 regimen versus the CoronaVac/CoronaVac one but also supports the results of a growing number of studies reporting the superiority of mix-and-match vaccine regimens in COVID-19 [24,25,26].

Interestingly, a mix-and-match BNT162b2/CoronaVac has already been tested but in a reversed way. A study from Hong Kong compared the immunogenicity of three strategies, homologous BNT162b2, heterologous vaccine (mRNA vaccine first dose then boost with inactivated vaccine), and homologous CoronaVac [27]. The results are consistent with our data since the authors showed significantly higher immunogenicity after the BNT162b2/CoronaVac regimen compared to the CoronaVac/CoronaVac group [27]. We should note that this study was an unblinded observational one. The participants were able to choose the type of vaccination according to their personal wishes and were notified of the collection of blood at baseline, day 21 (BNT162b2), or day 28 (CoronaVac and mix-and-match vaccine) [27].

The lack of data about the clinical efficacy of the mix-and-match CoronaVac/BNT162b2 regimen should be raised. In fact, no extended close follow-up was done beyond the study endpoint to observe longer-term vaccine effects. Nevertheless, all study participants were asked to report to the Principal Investigator any event that occurs after the trial comes to its planned end. They also have the right to be tested for free if they show suggestive symptoms of COVID-19, but no one has called to date. One way to circumvent this limitation was to assess the neutralizing antibody titers since they have been correlated to protection against SARS-CoV-2 infection [28]. The difference in neutralizing antibody levels identified in our study could thus be translated into substantial differences in vaccine effectiveness. Accordingly, a recent study showed that the mix-and-match CoronaVac/BNT162b2 manifested a higher protection level against infection compared to the homologous CoronaVac regimen in all tested times (from d15 to d90) [29]. According to this study, the CoronaVac/BNT162b2 regimen was not only superior in terms of efficacy to the CoronaVac homologous regimen but also to that of the BNT162b2/BNT162b2 regimen. Thus, CoronaVac/BNT162b2 vaccination could also be an alternative to the BNT162b/BNT162b2 regimen. Interestingly, this mix-and-match could improve long-term immunogenicity and efficacy since Lim et al. showed recently that a CD4 response elicited by inactivated virus vaccines seems to be more persistent over time than this elicited by mRNA vaccines [30]. Accordingly, Li et al. hypothesized that the T helper pool primed by the inactivated vaccine could be activated upon mRNA vaccination, thus facilitating the building of a stronger immune response and memory [31].

Moreover, it should be noted that our study predominantly recruited participants among workers (77%). Indeed, as mentioned above, the participants were recruited from the vaccination center of Ariana city but also from different companies (namely, Géant supermarket, Leoni factory or the Tunisian Electricity and Gas Company) since we have focused on the importance of social inclusion in health research to get the population adhesion to the vaccination policy. While this approach is appropriate for conducting a trial, it may limit the ability to generalize our results due to the risk of higher potential viral transmission in a confined workspace. Thus, future research using samples drawn from the general population is needed to test the robustness of our findings.

One last limitation of the study is the high number of people with baseline anti-S-RBD antibodies pointing out the presence of a history of asymptomatic COVID-19, although participants with a reported history of COVID-19 infection were ruled out. This could be explained by the wide SARS-CoV-2 virus circulation in the country through successive waves prior to the trial period, a situation that might have led to several asymptomatic infections [32]. This also explains the high titers of neutralizing antibodies (>85%) found in both groups after the second dose of the vaccine. Despite this limitation, the differences reported in our study were quite significant between both groups supporting the superiority of the mix-and-match regimen.

Regarding the side effects that occurred within 30 days after the second dose of vaccination, although they were significantly more frequent in the CoronaVac/BNT162b2 group compared to the CoronaVac/CoronaVac group, no severe events were noted in both groups. Collectively, our data not only support the superiority of the CoronaVac/BNT162b2 mix-and-match regimen but also demonstrate its better safety.

Our study is the first clinical trial demonstrating the superiority in terms of immunogenicity of the mix-and-match CoronaVac/BNT162b) regimen versus the homologous CoronaVac/CoronaVac regimen. Our results strongly support the possibility of using this regimen while ensuring good immunogenicity and tolerance safety. One of the perspectives of this study is to assess other potential correlates of protection, such as T cells or antibody-dependent cellular cytotoxicity, and to assess neutralizing potential towards the different COVID-19 new variants which increasingly escape the immune response, in particular the humoral one. Further extensive research confirming a longer duration of protection with the mix-and-match CoronaVac/BNT162b2 could place this latter as a prototype of choice in COVID-19 vaccination. It could even represent a good alternative for people reluctant to have iterative doses of vaccine. Moreover, mix-and-match regimens should be considered, under certain circumstances, as an alternative to address vaccine shortage and accelerate the national vaccine rollout plan. This trial could represent a model to be adopted as a vaccine regimen in a possible future pandemic involving new pathogens.

## Figures and Tables

**Table 1:** Baseline characteristics of the study population by vaccine protocol.

	Homologous Group (CoronaVac/CoronaVac)	Mix-and-Match Group (CoronaVac/Pfizer)	Total	*p*-Value
**Number of participants**	99	100	199	
**Sex,** *N* (%)				NS
Male	35(35.4)	45(45.0)	80(40.2)	
Female	64(64.6)	55(55.0)	119(59.8)	
**Age,** years				NS
Mean ±SD	32.3 ± 9.2	31.8 ± 7.7	32.0 ± 8.5	
Median (Min-Max)	31(18–60)	32 (18–48)	32(18–60)	
**Comorbidities,** *N* (%)				NS
Yes	29 (29.3)	21 (21.0)	50 (25.1)	
No	70 (70.7)	79 (79.0)	149 (74.9)	
**Tobacco use,** *N* (%)				NS
Yes	33 (33.3)	36 (36.0)	69 (34.7)	
No	66 (66.7)	64 (64.0)	130 (65.3)	
**Alcohol use,** *N* (%)				NS
Yes	11 (11.1)	16 (16.0)	27 (13.6)	
No	88 (88.9)	84 (84.0)	172 (86.4)	
**Study site,** *N* (%)				NS
Vaccination center	22 (22.2)	24 (24.0)	46 (23.1)	
Leoni factory	48 (48.5)	43 (43.0)	91 (45.7)	
Geant supermarket	21 (21.2)	24 (24.0)	45 (22.6)	
STEG head office	8 (8.1)	9 (9.0)	17 (8.5)	
**Neutralizing anti-SARS CoV-2** *N* (%)				NS
Positive	45 (45.5)	49 (49·0)	94 (49.0)	
Negative	54 (54.5)	51 (51·0)	105 (51.0)	
**Neutralizing anti-SARS CoV-2** (Median, IQR)	25 (0–79)	28 (0–70)	27 (0–77)	NS
**Anti-N protein IgG** *N* (%)				
Positive	53 (53.5)	58 (58.0)	111 (55.8)	NS
Negative	44 (44.4)	39 (39.0)	83 (41.7)	
**Anti-spike IgG,** *N* (%)				
Positive	61 (61.6)	73 (73.0)	134 (67.3)	NS
Negative	38 (38.4)	27 (27.0)	65 (32.7)	
**Anti-spike IgG, UI/mL**				
Median (IQR)	26·6 (0–229.3)	61·2 (0–193.2)	47·9 (0–224.4)	NS

SD: Standard deviation; IQR: Interquartile range; NS: Non-significant comparison.

**Table 2:** Neutralizing antibody and anti-spike IgG antibody responses after the first and the second dose in the homologous regimen compared with the mix-and-match group.

SARS-CoV-2 Antibody Titers	CoronaVac/ CoronaVac Group	CoronaVac/ BNT162b2 Group	Total	*p*-Value
**Neutralization antibody** **% inhibition after the first dose**				
Median (IQR)	90 (19–96)	94 (39–96)	93 (24–96)	NS
**Neutralization antibody** **% inhibition after the first dose,** *N* (%)				
- Negative: <30	32 (32.3)	24 (24.0)	56 (28.1)	
- Low: (30–60)	5 (5.1)	2 (2.0)	7 (3.5)	
- Medium: (60–90)	11 (11.1)	9 (9.0)	20 (10.1)	
- High: ≥90	51 (51.5)	65 (65.0)	116 (58.3)	NS
**Anti-spike IgG after the first dose**				
Median (IQR)	827 (4–2109)	1187(225–2475)	943(6–2245)	NS
**Time since first dose (days)**				
Mean ± SD Median (Min-Max)	22.4 ± 3·9 21 (20–42)	23.1 ± 4.9 21 (20–42)	22.8 ± 4.4 21 (20–42)	NS
**Neutralization antibody** **% inhibition after the second dose**				
Median (IQR)	94 (81–96)	96 (95–97)	96 (90–97)	*p* < 0.0001
**Neutralization antibody** **% inhibition after the second dose** *N* (%)				
- Negative:<30	2 (2.0)	1 (1.0)	3 (1.5)	
- Low: (30–60)	9 (9.1)	0 (0.0)	9 (4.5)	
- Medium: (60–90)	28 (28.3)	9 (9.0)	37 (18.6)	
- High: ≥90	60 (60.6)	90 (90.0)	150 (75.4)	*p* < 0.0001
**Anti-spike IgG after the second dose**				
Median (IQR)	1190 (347–4964)	13,460 (2557–29,930)	3513 (732–19,158)	*p* < 0.0001
**Time since second dose (days)**				
Mean ± SD	37.7 ± 8.7	37.0 ± 9.9	37.4 ± 9.3	NS
Median (Min–Max)	35 (27–70)	35 (20–105)	35 (20–105)	

SD: Standard deviation; IQR: Interquartile range; NS: Non-significant comparison.

**Table 3:** Side effects occurred within 30 days after the second dose by booster vaccine regimen.

	CoronaVac/CoronaVac Group n (%)	CoronaVac/BNT162b2 Group n (%)	Total n (%)	*p*-Value
**Side effects**				
Any	32 (32.2)	58 (58.0)	109 (54.8)	*p* < 10^−3^
Severe	0 (0.0)	0 (0.0)	0 (0.0)	-
Injection site reaction	1 (1.0)	4 (4.0)	5 (2.5)	NS
Arm Soreness	8 (8.0)	22 (22.0)	30 (15.1)	0.006
Fever	10 (10.1)	20 (20.0)	30 (15.1)	0.05
Headache	4 (4.0)	16 (16.0)	20 (10.1)	0.005
Tiredness	9 (9.1)	10 (10.0)	19 (9.5)	NS

NS: Non-significant comparison.

## Data Availability

All data in this study is available in the main text.
